# An Uncommon Presentation of Guillain-Barré Syndrome With Lhermitte Sign

**DOI:** 10.7759/cureus.77194

**Published:** 2025-01-09

**Authors:** Madalena Santos, Sofia Martins, Duarte Graça, Miguel Carrilho, Alba Acabado

**Affiliations:** 1 Internal Medicine, Hospital De Santa Maria, Lisbon, PRT; 2 Internal Medicine, Centro Hospitalar Universitário Lisboa Norte, Lisbon, PRT

**Keywords:** acute inflammatory demyelinating polyradiculoneuropathy, areflexia, flaccid motor paralysis, lhermitte sign, women

## Abstract

Guillain-Barré syndrome (GBS) is a complex and potentially life-threatening disease, representing the most common cause of acute neuromuscular paralysis worldwide. Its diagnosis is primarily based on clinical findings, often complemented by electrophysiological studies and laboratory investigations. Therefore, knowledge of the clinical signs and symptoms is essential to make a prompt diagnosis and allow timely initiation of therapeutic interventions. Despite significant advancements in our understanding of this disease, many aspects of its nature are yet to be fully understood.

We present a compelling case that initially manifested with the Lhermitte sign, followed by the development of paresthesias and diminished muscle strength originating in the upper extremities and progressing in a cephalocaudal pattern. These initial findings suggest a pathology involving the cervical spinal cord or nerve roots. However, the patient’s evolving neurological deficits and subsequent diagnostic studies revealed an unexpected etiology: GBS.

This case highlights the importance of maintaining a broad differential diagnosis, even when confronted with seemingly characteristic clinical presentations. It also reveals the complexity of GBS, a disease that can present with potential atypical manifestations that can mimic other neurological disorders.

## Introduction

Guillain-Barré syndrome (GBS) represents an acute inflammatory demyelinating polyradiculoneuropathy that provokes a flaccid motor paralysis with areflexia, characterized by rapid progression and an ascending pattern, with or without sensory alterations [1]. It constitutes the primary cause of acute flaccid paralysis worldwide, with an incidence of one to two cases per 100000 inhabitants annually, peaking between 50 and 70 years of age, and a female-to-male ratio of 1:1.5 [2].

In contemporary medical literature, the main precipitating events consist of infectious agents, vaccinations, and certain systemic diseases (lymphoma and lupus), all of which appear to play a crucial role in the pathogenesis of GBS [1]. Specifically, about 70% of GBS cases emerge one to three weeks following an acute infectious process, typically respiratory or gastrointestinal. The most common associated agents include* Campylobacter jejuni, Haemophilus influenzae, Mycoplasma pneumoniae*, cytomegalovirus (CMV), Epstein-Barr virus (EBV), varicella-zoster virus (VZV), and human immunodeficiency virus (HIV) [1].

Regarding immunization processes, there have been cases of GBS associated with the "swine flu" vaccine (A/New Jersey/76 influenza), the recombinant zoster vaccine (RZV), and the adenovirus-vector SARS-CoV-2 vaccines [2]. Notably, there have been documented cases of GBS following stem cell transplantation in patients receiving tacrolimus for graft-versus-host disease (GVHD) prophylaxis, in patients undergoing treatment with immune checkpoint inhibitors, and even in patients after anti-B-cell maturation antigen (BCMA) chimeric antigen receptor T-cell (CAR-T) therapy [2,3]. Nevertheless, the actual risk of developing GBS has not been ascertained in controlled studies.

The pathophysiology of GBS is characterized by a complex autoimmune cascade initiated by specific triggers, where involved antigens share structural similarities with components of peripheral nerves, leading to an immune-mediated disruption of axons and/or myelin [2,4].

GBS can have a diversity of manifestations, according to the pattern of neurological damage, ranging from: 1. A predominant demyelinating process, where the immune response targets the myelin sheath surrounding peripheral nerves, causing impaired nerve conduction and the classic ascending paralysis associated with GBS; 2. Axonal damage, especially to motor neurons; and 3. A more extensive form of axonal damage that can affect both motor and sensory nerve fibers [4,5].

The main clinical features of GBS consist of an acute, rapidly progressive flaccid paralysis (ascending and symmetrical) and decreased or absent deep tendon reflexes, with or without sensory deficits [2]. Following exposure to an immunological trigger, there's a characteristic latency period of approximately 10 to 14 days, during which the immune-mediated processes begin. Afterward, the hallmark symptoms begin to manifest, evolving gradually and progressively, followed by a rapid evolution with the nadir of neurological deficits usually reached within four weeks of onset [1,2]. The severity of this disease can be such that approximately 30% of patients require mechanical ventilation due to respiratory muscle weakness [6].

Autonomic dysfunction is also a significant concern in GBS, affecting a substantial proportion of patients. This dysfunction can manifest through various symptoms, including orthostatic hypotension, sweating abnormalities, and gastrointestinal issues [7]. Besides the typical temporal history and clinical manifestations described above, the diagnosis of GBS also encompasses CSF albuminocytological dissociation, with high protein level and normal white cell count (WCC); electrophysiological studies with either a slowing of conduction velocity, prolonged distal motor latencies and delayed F waves, or reduced sensory and/or motor nerve action potentials, along with absent H-reflexes; and positive ganglioside antibodies (both IgG and IgM isotypes) [1,2].

One should emphasize that normal CSF protein is common during the first week of the disease and may be normal in the second week after the onset of symptoms. Also, the absence of ganglioside antibodies does not exclude GBS [2]. The list of differential diagnoses can be extensive and can be classified according to the location in the nervous system: CNS: inflammation/infection of the brainstem and spinal cord (acute transverse myelitis); anterior horn cells: acute flaccid myelitis caused by infectious agents; nerve roots: infection or toxicity, as in acute intermittent porphyria; peripheral nerve disease: chronic inflammatory demyelinating polyradiculoneuropathy (CIDP), eosinophilic granulomatosis with polyangiitis (EGPA); neuromuscular junction: myasthenia gravis, Lambert-Eaton myasthenic syndrome [8].

Currently, treatment relies on modifying therapies, mainly intravenous immunoglobulin (IVIg) and plasmapheresis [1]. A less common treatment known as immunoabsorption (IA), the selective removal of IgG from the blood by passing separated plasma through an absorption column, is also described in scientific literature, though it is not recommended as first or second-line therapy [2].

GBS is a treatable condition where most patients recover without complications [2]. Nevertheless, up to 10% of patients may have a severe disability and can even become ventilator-dependent [2]. Mortality is estimated to be around 3%-13%, with the most common causes being respiratory failure, pneumonia, cardiac arrest, and autonomic dysfunction [9]. Negative prognostic factors include older age, severe weakness on admission, and the need for mechanical ventilation [9].

## Case presentation

A 43-year-old woman with a medical history of essential hypertension controlled with a calcium channel blocker, angiotensin II receptor antagonist, and thiazide diuretic, without any other relevant past medical history, was brought to the Emergency Department (ED) with progressive and incapacitating paresthesias and decreased muscle strength. Initially, the patient experienced Lhermitte's sign, a transient, electric shock-like sensation originating in the neck and radiating down the spine, triggered by cervical flexion, and it resolved within a fortnight. Afterward, she developed paresthesias in her left upper extremity, accompanied by a concomitant decrease in muscle strength. This sensorimotor disturbance rapidly evolved over the course of a week, extending symmetrically to involve the contralateral upper limb and both lower extremities, with a fluctuating course. The escalating severity of her condition was marked by an episode of a fall from standing height due to the worsening muscle weakness, without loss of consciousness or cranial trauma, prompting her visit to the ED, almost a month after the initial symptom (Lhermitte's sign). The patient was unaware of any recent infectious process and denied recent immunization.

Upon clinical assessment, the patient's vital parameters were within physiological limits. On neurological examination, notable findings included symmetrical decreased strength in the upper limbs, in finger extension (G4/5), and also in the lower limbs, in hip flexion (G4/5), with remaining segments 5/5. Plantar responses were flexor bilaterally. The deep tendon reflexes were abolished in all four limbs. Sensory testing revealed painful hypoesthesia in all four limbs, while trunk sensibility remained intact. Proprioception was preserved, but vibratory sensation was diminished in the lower extremities up to the knee level. Coordination testing excluded appendicular ataxia or dysdiadochokinesia, and the patient had an unstable gait, with possible ataxia, and a positive Romberg sign. No other abnormalities were found during the physical examination.

Following consultation with the neurology service, the patient was admitted to the Internal Medicine Department for an etiological investigation. On admission, brain and cervical spine MRIs were performed, excluding any significant abnormalities, along with an electrocardiographic assessment and chest radiography. An electromyographic study revealed a decrease in conduction velocities and delayed F waves, features compatible with marked, primarily demyelinating sensory-motor polyneuropathy.

The analytical evaluation yielded no significant abnormalities and excluded any metabolic or endocrine abnormalities, as well as infectious processes. Antibodies against myelin-associated glycoprotein (Anti-MAG) and ganglioside antibodies tested negative, as did the laboratory markers for acute porphyrias.

In addition to the etiological investigation described above, a lumbar puncture was performed with extraction of crystal-clear fluid. The cytochemical analysis revealed findings, as shown in Table 1.

**Table 1:** Laboratory parameters analyzed in the CSF CSF: cerebrospinal fluid

Laboratory parameters		Value (units)	Reference value
CSF	Color	Clear	-
	Lymphocytes	2/mm^3^	<5
	Glucose	65 mg/dL	40-70
	Protein	76.9 mg/dL	15-45
	Albumin	380 mg/L	0-350
Serum	Glucose	81 mg/dL	70-110
	Albumin	3.8 g/dL	3.5-5.2
CSF/Serum albumin ratio		100	<9

There was also a negative bacteriological and serological evaluation for Herpes simplex types 1 and 2, and VZV. No oligoclonal bands were detected, nor was there an increase in the CSF/serum IgG ratio.

Given the findings of the etiological investigation, with regards to the lumbar puncture and electromyogram, and the clinical presentation of the patient, a presumptive diagnosis of acute inflammatory polyradiculoneuropathy was made, and treatment with IVIG was started (0.4g/kg/day). The patient underwent IVIG therapy for five days, with a marked improvement in neurological symptoms, particularly in gait, two weeks after the first dose. Upon discharge, the neurological examination revealed that motor function was preserved with full strength (5/5) in all segments, except for bilateral great toe extension, which was slightly reduced (4+/5); normal deep tendon reflexes in the upper extremities (2+), while patellar and Achilles reflexes were diminished bilaterally; flexor plantar responses; sensory testing demonstrated hypoesthesia with a high sock distribution in the left lower extremity, and pallhypesthesia in the left great toe; intact proprioception in all four limbs, and independent ambulation without a widened base.

Therefore, the patient was diagnosed with GBS and exhibited a favorable clinical evolution, albeit with residual neurological deficits as previously described.

## Discussion

The patient's clinical journey began with the onset of Lhermitte's sign, a transient, electric shock-like sensation that originated in the neck, triggered by cervical flexion, radiated down the spine, and, in this case, resolved in two weeks. This phenomenon is typically associated with demyelinating conditions, such as multiple sclerosis (MS), where damage to the myelin sheath disrupts normal nerve conduction, leading to abnormal sensory experiences when the neck is flexed [10].

Following the resolution of Lhermitte's sign, the patient began to experience paresthesias, abnormal sensations such as tingling or prickling, in her left upper extremity. This was accompanied by a notable decrease in muscle strength, indicating a potential compromise in both sensory and motor pathways. Over the course of one week, these symptoms rapidly progressed and extended symmetrically to involve the contralateral upper limb and both lower extremities. The evolving nature of these symptoms suggests an underlying neuropathic process that may be characteristic of inflammatory or demyelinating conditions [2].

The severity of the patient's condition escalated, culminating in an episode where she fell from a standing position due to worsening muscle weakness. Importantly, this incident occurred without loss of consciousness or cranial trauma, which helps to differentiate it from syncope-related falls. The timing of this fall, almost a month after the initial presentation of Lhermitte's sign, highlights the progressive nature of her neurological deficits.

Throughout this period, the patient reported no awareness of recent infections or immunizations that could explain her symptoms. This lack of identifiable triggers further complicated the diagnosis, indicating a prompt necessity for a thorough investigation into potential underlying causes.

Upon initial evaluation, the denial of any potential triggers together with the presence of Lhermitte's sign, and the development of paresthesias with decreased muscle strength in a clockwise progression, strongly suggested a demyelinating condition with spinal cord involvement, such as MS, neuromyelitis optica, acute disseminated encephalomyelitis, acute transverse myelitis, and optic neuritis [11].

The findings from the CT and MRI scans, along with the results of the neurological examination, effectively ruled out this initial hypothesis. More specifically, the presence of symmetrical upper and lower limb flaccid paralysis, as well as bilateral flexor plantar responses, indicating intact corticospinal pathways, and the abolition of deep tendon reflexes are all indicative of lower motor neuron involvement or peripheral nerve damage. Such findings are consistent with the pathophysiology of GBS, where demyelination and axonal damage impair normal reflex pathways [12].

Sensory examination revealed painful hypoesthesia in all four limbs, indicating compromised sensory nerve function. Interestingly, trunk sensation remained intact, suggesting that the sensory pathways from the trunk were unaffected. Proprioception was preserved; however, vibratory sensation was diminished in the lower extremities up to the level of the knees. This differential sensory loss points toward length-dependent neuropathy, which is a common presentation of GBS.

Coordination assessments ruled out appendicular ataxia and dysdiadochokinesia, indicating that cerebellar function was likely intact. However, the patient demonstrated an unstable gait and possible ataxia, alongside a positive Romberg sign. The positive Romberg test suggests impaired proprioceptive input necessary for maintaining balance, further supporting the notion of peripheral nerve involvement.

Overall, these neurological examination findings provide critical insights into the extent and nature of the peripheral nerve dysfunction characteristic of GBS. The integration of the clinical manifestations, the electrophysiological findings, and the characteristic cerebrospinal fluid (CSF) profile, particularly albuminocytological dissociation, were all crucial for establishing a definitive diagnosis of GBS.

## Conclusions

In summary, this case illustrates a complex interplay between sensory and motor dysfunctions indicative of a demyelinating or inflammatory process affecting the peripheral nervous system. The presence of Lhermitte's sign serves as an important initial clinical marker, while the subsequent development of paresthesias and muscle weakness underscores the need for comprehensive neurological evaluation and timely intervention to address the evolving symptoms. This case highlights the critical importance of clinicians maintaining a high index of suspicion for GBS, even in the presence of atypical neurological manifestations, such as Lhermitte's sign. While Lhermitte's sign is typically associated with conditions such as MS or cervical myelopathy, its occurrence as an initial symptom in GBS is exceptionally rare and not well-documented in current literature. 

However, given the diverse and sometimes atypical presentations of GBS, including unusual sensory symptoms, this unique manifestation warrants consideration in the differential diagnosis. This case emphasizes the importance of recognizing atypical presentations in GBS to ensure prompt diagnosis and appropriate management. Physicians should be aware that GBS can present with diverse and atypical symptoms, such as in this case. A thorough neurological assessment, coupled with an understanding of the potential for atypical presentations, is crucial for early diagnosis and prompt initiation of appropriate treatment, which can significantly impact patient outcomes in GBS.
